# Tetrandrine Suppresses Cancer Angiogenesis and Metastasis in 4T1 Tumor Bearing Mice

**DOI:** 10.1155/2013/265061

**Published:** 2013-05-25

**Authors:** Jian-Li Gao, Xing Ji, Tong-Chuan He, Qi Zhang, Kai He, Yan Zhao, Su-Hong Chen, Gui-Yuan Lv

**Affiliations:** ^1^Zhejiang Chinese Medical University, Hangzhou, Zhejiang 310053, China; ^2^Molecular Oncology Laboratory, Department of Surgery, The University of Chicago Medical Center, Chicago, IL 60637, USA; ^3^Wenzhou Medical College, Wenzhou, Zhejiang 325035, China; ^4^The First Affiliated Hospital of Zhejiang University, Hangzhou, Zhejiang 310009, China

## Abstract

Metastasis remains the most deadly aspect of cancer and still evades direct treatment. Thus, there is a great need to develop new treatment regimens to suppress tumor cells that have escaped surgical removal or that may have already disseminated. We have found that tetrandrine (TET) exhibits anticolon cancer activity. Here, we investigate the inhibition effect of TET to breast cancer metastasis, angiogenesis and its molecular basis underlying TET's anticancer activity. We compare TET with chemotherapy drug doxorubicin in 4T1 tumor bearing BALB/c mice model and find that TET exhibits an anticancer metastatic and antiangiogenic activities better than those of doxorubicin. The lung metastatic sites were decreased by TET, which is confirmed by bioluminescence imaging *in vivo*. On the other hand, laser doppler perfusion imaging (LDI) was used for measuring the blood flow of tumor in 4T1-tumor bearing mice. As a result, the local blood perfusion of tumor was markedly decreased by TET after 3 weeks. Mechanistically, TET treatment leads to a decrease in p-ERK level and an increase in NF-**κ**B levels in HUVECs. TET also regulated metastatic and angiogenic related proteins, including vascular endothelial growth factor, hypoxia-inducible factor-1**α**, integrin **β**5, endothelial cell specific molecule-1, and intercellular adhesion molecule-1 *in vivo*.

## 1. Introduction

Tetrandrine (TET), a bisbenzylisoquinoline alkaloid isolated from the dried root of *Stephania tetrandra* (or *hang fang ji*) of the Menispermaceae, is a bioactive alkaloid with a molecular weight of 622.76 g/mol. Many reports indicated that TET exhibits very broad pharmacological actions, including immunomodulating, antihepatofibrogenetic, antiinflammatory, antiarrhythmic, antiportal hypertension, anticancer, and neuroprotective activities [1]. The beneficial effects of TET on tumor cell cytotoxicity and radiosensitization, multidrug resistance, normal tissue radioprotection, and angiogenesis are most promising and deserve great attention [2, 3]. Several investigations indicated that TET generally presents its anticancer effects in the micromolar concentrations on clone, leukemia, bladder, hepatoma, and lung cancer *in vitro* [4–8].

In our previous research, we have found that TET exhibits significant anticancer activity in colon cancer line HCT116. Mechanistically, the inhibitory effect of TET on colon cancer cells may be at least in part mediated by targeting *β*-catenin activity, and the sensitivity of cancer cells to TET may be determined by the functional status of *β*-catenin [9].

Nevertheless, with the development of clinical surgical treatment, the growth of primary tumor was no longer the critical element influencing the overall survival in cancer patients. Prevention of metastasis and more effective treatment of cancer metastasis are necessary for cancer therapy. Thus, antimetastasis therapy that targets tumor cells escaped surgical removal or already disseminated, and tumor angiogenic process has a better chance of success. Although several rational lines of evidence support the application of TET as an anticancer metastatic agent [10], the cellular mechanisms underlying the antiangiogenic and antimetastatic effects of TET activation in tumor cells remain elusive.

In this study, we use a mouse model for stage IV breast cancer (4T1 tumor bearing BALB/c mice model) for evaluating the antimetastatic effect of TET in lower concentration (10 mg/kg/d, which could not change the growth of tumor mass). Meanwhile, laser Doppler imager (LDI) was used for measuring blood perfusion of the tumor bearing area, which could measure the local blood flow and vascular network and reflect the angiogenic activity of the tumor bearing mice indirectly. Our results show that TET could significantly inhibit endothelial cell (EC) proliferation, adhesion, migration, invasion, and tube formation by targeting angiogenic factors, namely vascular endothelial growth factor (VEGF) and hypoxia-Inducible factor-1*α* (HIF-1*α*), as well as adhesion factors, such as integrin *β*5, endothelial cell specific molecule-1 (ESM-1), and intercellular adhesion molecule-1 (ICAM-1), and by interfering with the ERK pathway, leading to the suppression of tumor metastasis and tumor angiogenesis.

## 2. Materials and Methods

### 2.1. Cell Culture and Animals

Mouse breast cancer 4T1 and HEK-293 cells were purchased from American Type Culture Collection (ATCC, Manassas, VA, USA) and grown in the DMEM (Invitrogen, Carlsbad, CA, USA) supplemented with 10% FBS (Hyclone, Logan, UT, USA) and 50 U penicillin/streptomycin. Human umbilical vein endothelial cell line (HUVEC) was immortalized as described in [11] and maintained in RPMI 1640 medium (Invitrogen, Carlsbad, CA, USA) supplemented with 15% FBS, 2 mM L-glutamate, 100 U/mL penicillin and 100 *μ*g/mL streptomycin. All cells were cultured at 37°C in a 5% CO_2_ incubator.

Female BALB/c mice (4-5 weeks old) were purchased from Shanghai Lab. Animal Research Center and maintained at the animal facility of Experimental Animal Research Center of Zhejiang Chinese Medical University. All procedures were performed according to protocols following the guidelines for the Use and Care of Laboratory Animals published by the US National Institutes of Health (NIH Publication no. 85–23, revised 1996).

### 2.2. Chemicals and Drug Preparations

TET and doxorubicin (Dox) were purchased from Sigma-Aldrich (St. Louis, MO, USA). These compounds were dissolved in DMSO to make stock solutions and were kept at −20°C as aliquots. The solution was diluted with Milli-Q water into 2 mg/mL and ultrasonificated into a fine suspension before *in vivo* use. 0.1% DMSO was used in vehicle group in *in vitro* assays.

### 2.3. Establishment of Stably Tagged 4T1-Luc Cell Lines

The 4T1 cells were stably transduced with firefly luciferase by using a retroviral vector expressing firefly luciferase as described in [12]. Briefly, recombinant retrovirus was packaged in HEK-293 cells by cotransfecting cells with pSEB-Luc and pAmpho packaging plasmid using LipofectAMINE (Invitrogen). Pooled stable cells were selected with blasticidin S (6 *μ*g/mL) for 7 days. The firefly luciferase activity was confirmed by using Promega's Luciferase Assay kit (Promega, Madison, WI, USA).

### 2.4. MTT Proliferation Assay

A modified MTT assay was used to examine the cell proliferation as described in [13]. Briefly, iHUVEC (1 × 10^4^ cells/well, 50–70% density) cells were seeded in 96-well plates. Drugs were added to the cells at variable concentrations or solvent control (0.1% DMSO). At 48 h after treatment, 15 *μ*L MTT dye solution was added to each well and incubated for additional 4 h. Subsequently, the cell culture medium was removed, and 100 *μ*L/well DMSO was added to dissolve formazan crystals in a humidified atmosphere overnight. Absorbance at 570 nm was measured using a 96-well microplate reader.

### 2.5. Crystal Violet Viability Assay

Crystal violet assay was conducted as described in [14]. Experimentally, iHUVEC cells were treated with drugs. At 24 h after treatment, cells were carefully washed with PBS and stained with 0.5% crystal violet formalin solution at room temperature for 20–30 min. The stained cells were washed with tape water and air-dried for taking macrographic images. For quantitative measurement, the stained cells were dissolved in 10% acetic acid (1 mL per well for 12-well plate) at room temperature for 20 min with shaking. Absorbance at 570–590 nm was measured.

### 2.6. Cell Adhesion Assay

For the cell adhesion model, HUVECs and 4T1 cells were used to study the adhesive ability between two different kinds of cell types. Briefly, HUVECs (2 × 10^4^ each well) were grown to confluence on fibronectin-coated wells of 96-well plates. The plates were blocked with Hank's balanced salt solution (HBSS) containing 1% bovine serum albumin (BSA) (HBSS-BSA) for 30 min before the adhesion assay. BSA-coated wells serve as a negative control.

The 4T1 cells were trypsinized and suspended in HBSS-BSA and then labeled with 10 *μ*L Hoechst 33258 for 30 min at 37°C followed by washing with HBSS-BSA. The labeled 4T1 cells were then suspended in HBSS-BSA to a final density of 4.0 × 10^5^ cells/mL, and different dosages of TET were added. Cell suspension (100 *μ*L/well) was incubated with HUVECs at 37°C for 30 min. Cultures were carefully washed three times with PBS to remove nonadherent cells. Three random views were photographed in each well at 100x magnification with an inverted fluorescence microscope (Olympus Corporation, Japan). The image was analyzed with Image-Pro Plus 6 software (Media Cybernetics, USA).

### 2.7. Cell Migration Assay

A wound-healing model was used for evaluation of cell migration ability [15]. Cells treated with 0.1% DMSO were used as the vehicle control. Three random views along the scraped line were photographed in each well at 100x magnification before and after 10 h drug treatment with an inverted fluorescence microscope. The image was analyzed with Image-Pro Plus 6 software. Average scraped width of each well was measured and compared with control.

### 2.8. Boyden Chamber Transwell Cell Invasion Assay

Cell invasive ability was measured on a transwell system with a polycarbonate membrane (8 *μ*m pores) as previously described in [15]. The upper and lower sides of the membrane were precoated with 1 : 30 (v/v) and 1 : 100 (v/v) matrigel, respectively. The iHUVECs (50,000 cells) were seeded into culture inserts. Low-serum medium containing different concentrations of TET was added into the plate wells. After 12 h, the inserts were washed with PBS; upper surface cells were removed by cotton swabs and the lower side was fixed in 3.7% paraformaldehyde. The invasive cells were then stained with propidium iodide (PI) and mounted on microscope slides. Images were captured at 200x magnification with an inverted fluorescence microscope. Invasive cells were quantified by Image-Pro Plus 6 software. The number of migrate cells per fields was determined by averaging nine randomly counted fields.

### 2.9. Tube Formation Assay

The effects of the drugs on HUVEC differentiation were examined by their *in vitro* tube formation ability on matrigel [15]. HUVECs were harvested and diluted to 2 × 10^5^ cell/mL in low-serum medium (0.5% FBS) containing 20 ng/mL Vascular Endothelial Growth Factor (VEGF) and different concentrations of drugs. The cells were then seeded onto 1 : 1 matrigel (v/v) coated 24-well plates at 37°C for 8 h. Cells treated with 0.1% DMSO were used as the vehicle control. The branch points of the capillary-like tubes were counted under light microscopy (100x field).

### 2.10. Cell Cycle Analysis by Flow Cytometry

Flow cytometry was used for quantitatively detecting the cell-cycle distribution [16]. Cells (1 × 10^5^/well) were plated into 6-well plates 1 day before treatment with TET at various concentrations. After treatment for 24 and 48 h, cells were harvested, washed with PBS, fixed in cold 3.7% paraformaldehyde overnight at 4°C for at least 2 h, and stained with 50 ng/mL PI in the presence of 200 *μ*g/mL RNase A by incubation at 37°C for at least 30 min. The stained cells were analyzed by flow cytometry (Becton-Dickinson). The red fluorescence (PE) representing the DNA content was collected through a 585 nm filter. Data were analyzed using Mod Fit LT 3.0 software.

### 2.11. Protein Extraction and Western Blotting Analysis

Western blotting was performed as previously described in [17]. Briefly, cells were collected and lysed in RAPI buffer. After treatment on ice for 30 min, cell lysates were clarified by centrifugation at 11,419 ×g for 20 min at 4°C to remove cell debris and the protein content was measured using a BCA protein assay kit (Beyotime, Jiangsu, China). Cleared total cell lysate was denatured by boiling, and aliquots of the lysates were loaded onto a 10% gradient SDS-PAGE. After electrophoretic separation, proteins were transferred to an Immobilon-P membrane. Membrane was blocked with SuperBlock Blocking Buffer and probed with the primary antibody, anti-NF-*κ*B (Santa Cruz Biotechnology, Santa Cruz, CA, USA), anti-ERK1/2, and p-ERK1/2 (Cell Signaling Technology, Vancouver, Canada), followed by incubation with a secondary antibody conjugated with biotin. Then the PVDF membrane was incubated with streptavidin HRP. The proteins of interest were detected by using SuperSignal West Pico Chemiluminescent Substrate kit.

### 2.12. Gelatin Zymography

Gelatin zymography was performed on 7.5% polyacrylamide gels containing 0.1% gelatin as previously described in [15]. Cells were treated as indicated in 0.5% FBS RPMI1640 (containing 20 ng/mL VEGF) for 24 h. The cell culture medium was then centrifuged at 350 ×g for 4 min at 4°C, and the total protein of the supernatant was normalized with BCA protein assay kit. The supernatant was mixed with 5x nonreducing sample buffer and loaded onto 10-well gels (20 *μ*L/sample), and electrophoresis was performed at 100 V for 1.25 h. After electrophoresis, the gel was rinsed with 1x renaturing buffer for 1.5 h at room temperature. The buffer was then changed to 1x developing buffer and incubated for 48 h at 37°C. Gelatin gel was stained with Coomassie blue and then destained with 10% acetic acid. The unstained bands correspond to the areas of gelatin digestion.

### 2.13. 4T1 Tumor Bearing Mice Model

Female BALB/c mice (4 weeks old, 18–20 g, 10 mice per group) were used. Subconfluent 4T1-Luc cells were harvested and resuspended in PBS to a final density of 1 × 10^7^ cells/mL. Before injection, cells were resuspended in PBS and analyzed by 0.4% trypan blue exclusion assay (viable cells, >90%). For cancer cell injection, approximately 5 × 10^5^ 4T1-Luc cells in 100 *μ*L of PBS were injected into the mammary fat pad (MFP) of each mouse using 27 gauge needles [18]. At 48 h after tumor cell injection, TET was administered at 10 mg/kg body weight to mice once every 2 days orally, and Dox was administered by intraperitoneal injection at 1 mg/kg/2 days to mice as a positive control.

### 2.14. Xenogen Bioluminescence Imaging

Small animal whole-body optical imaging was carried out as described previously in [9]. In brief, mice were anesthetized with isoflurane attached to a nose-cone mask equipped with Xenogen IVIS 200 imaging system (Caliper Life Sciences, Hopkinton, MA, USA) and subjected to imaging weekly after MFP injection.

For imaging, mice were injected intraperitoneally with D-luciferin sodium salt (Gold Biotechnology, St. Louis, MO, USA) at 100 mg/kg body weight in 0.1 mL of sterile PBS. Acquired images were obtained by superimposing the emitted light over the grayscale photographs of the animal. Quantitative analysis was done with Xenogen's Living Image V2.50.1 software as described previously in [14]. Animals were taken *in vivo* images for both untreated and treated groups and sacrificed after 4 weeks. Tumor, lung, and vascular samples were retrieved for histological examination.

### 2.15. High-Resolution Laser Doppler Perfusion Imaging

Microvascular blood flow was assessed by laser Doppler with a moorFLPI V2.1 software (Moor instruments Ltd, UK) [19, 20]. Mouse hair was carefully removed and mice were anesthetized with isoflurane attached to a nose-cone mask. With a distance of 10 cm between the scanner and the skin surface, three examined areas (1.4∗1.4 cm) were chosen so that tumor (Flux 1), adjacent healthy skin around tumor (Flux 2), and the heart of mice (Flux 3) were covered. The laser beam is reflected by the erythrocytes, which allows recording of the returning signal by a detector positioned in the scanner head and thus conversion to an electrical signal, proportional to the tissue perfusion. The underlying intensity of perfusion values is expressed on a scale of different colours extending from blue (low perfusion values) over green and yellow to red (highest perfusion values). The related perfusion values were calculated as follows: perfusion rate (tumor) = *F*1/*F*3∗100%; perfusion rate (vascular) = *F*2/*F*3∗100%. 

### 2.16. Histological Evaluation and Immunohistochemical Staining

Retrieved tumor tissues were fixed in 10% formalin and embedded in paraffin. Serial sections of the embedded specimens were stained with hematoxylin and eosin. For immunohistochemical staining, slides were deparaffinized and then rehydrated in a graduated fashion [21]. The deparaffinized slides were subjected to antigen retrieval and probed with anti-ICAM-1, anti-HIF-1*α*, anti-integrin *β*5, anti-ESM-1, or anti-VEGF antibody (Santa Cruz Biotechnology) or isotype IgG control, followed by incubation with biotin secondary antibodies and streptavidin-horseradish peroxidase. The presence of the expected protein was visualized by DAB staining and examined under a microscope. Stains without the primary antibody were used as negative controls.

### 2.17. Statistical Analysis

Data were expressed as mean ± S.D. Statistical significances between vehicle group versus drug treatment groups were determined by one-way analysis of variance. The IC_50_ of the TET was calculated by SPSS software. A value of *P* < 0.05 was considered to be statistically significant.

## 3. Results

### 3.1. TET Exhibits a Significant Growth-Inhibitory Effect in HUVECs

**Figure 1:**
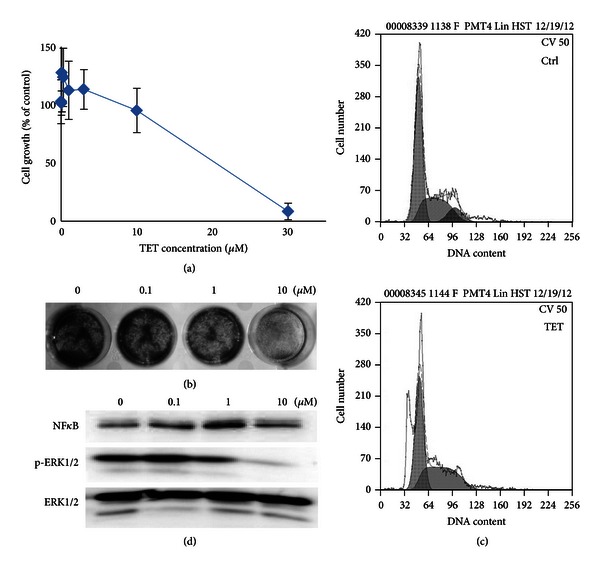
Antiproliferative activity of TET in HUVECs. (a) MTT assay. Subconfluent HUVECs were treated with indicated concentrations of TET for 48 h. The cells were then subjected to MTT assay. Each assay condition was done in triplicate (*n* = 12). (b) Crystal violet assay. HUVECs were treated with TET at the indicated concentrations for 24 h. The cells were subjected to crystal violet assay as described in Section 2. (c) Cell cycle analysis. Cell cycle distribution of HUVECs was analyzed by flowcytometry. Cells were treated with 1 *μ*M TET for 24 h and fixed, and then nuclear DNA was labeled with PI. Histogram display DNA content (*x*-axis [PE-A]: PI-fluorescence) versus cell counts (*y*-axis). (d) ERK, p-ERK, and NF*κ*B expressions in HUVECs. The expression of ERK, p-ERK, and NF*κ*B was detected by western blot.

To assess the antiangiogenic property of TET *in vitro*, we examined the inhibitory effects of TET on cell viability in HUVECs using MTT assay and crystal violet staining. As shown in Figures 1(a) and 1(b), TET (<10 *μ*M) does not have any remarkable effect on HUVEC proliferation. However, TET can significantly inhibit cell viability at a much higher concentration with a half-maximal inhibition at 16.76 *μ*M (by MTT assay) or 29.31 *μ*M (by crystal violet staining assay). To examine the possible mechanism behind TET's inhibition effect on HUVEC's proliferation, we performed cell cycle analysis by FACS, and the result revealed that when HUVECs were treated with TET for 24 h, TET in 10 *μ*M induced a depletion of cells in the G_2_-M phase, from 10.70% to 4.21%, and a concomitant accumulation of cells in S phase, from 34.17% to 38.35%. These data suggested that TET could arrest endothelial cell proliferation. Furthermore, we examined the effects of TET on NF-*κ*B, ERK1/2, and p-ERK1/2 expressions, and the results suggested that the inhibition of TET in HUVECs was related to the upregulation of NF-*κ*B and suppression of the phosphorylation of ERK1/2.

### 3.2. TET Inhibits Cell Adhesion, Cell Migration, and Cell Invasion of HUVECs

**Figure 2:**
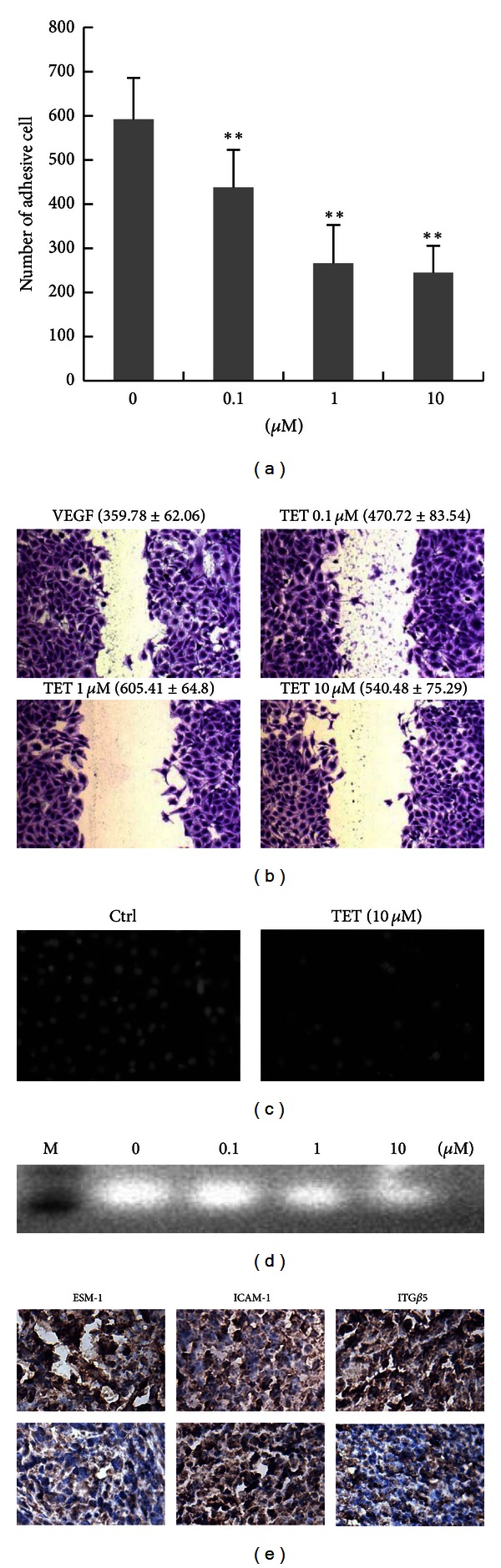
TET inhibits cell adhesion (a), migration (b), and invasion (c) in HUVECs. (a) Cell adhesion assay. HUVECs and 4T1 cells were used to study the cell adhesive ability. **P < 0.01 versus vehicle control. (b) Cell migration assay. Wound-healing model was used for evaluation of cell migration ability. Three random views were photographed along the scraped line in each well at 100x magnification. (c) Cell invasion assay. Cell invasive ability was measured with a transwell system with a polycarbonate membrane (8 *μ*m pores). Images were captured at 200x magnification. (d) Effects of TET on secretion of matrix metalloproteinase-2 (MMP-2). Gelatin zymography was carried out in an SDS-PAGE gel that contained 0.1% gelatin. (e) Effect of TET on ESM-1, ICAM-1, and integrin *β*5 expressions in tumor tissues. 4T1 tumor bearing mice (top panel) and TET treated tumor bearing mice (bottom panel) (magnification ×400).

The antiadhesion ability of TET between 4T1 and HUVEC cells was investigated. As a result, TET could suppress the 4T1 and HUVEC adhesion after 30 min treatment (Figure 2(a)). The adhesive cells were decreased to 245 compared with 592 of the control group. 

Moreover, the VEGF-induced migration of HUVEC cells was significantly suppressed by TET (Figure 2(b)). The migratory distance of HUVEC was significantly decreased by 10 *μ*M TET-contained medium when compared to that of control (*P* < 0.01). We further tested whether TET could affect chemotactic HUVECs invasion. Using the Boyden chamber transwell assay, we found that when HUVECs were treated with 10 *μ*M TET the numbers of migrated cells across the extracellular matrix protein-coated membranes significantly decreased (Figure 2(c)). Quantitatively, TET was shown to inhibit the numbers of migrated HCT116 cells by approximately 33% over that of the control treatment. 

Notably, this inhibitory effect of TET on EC invasion was potentially related to the activity of proteinases (MMPs). To determine the effect of TET on the production of proteinases by HUVEC, culture supernatants were collected and subjected to gelatin zymography. As shown in Figure 2(d), the presence of proteinases (MMPs) digested the gelatin-containing gel and resulted in a clear band at 66 kDa, which was assigned to MMP-2, and TET reduced the gelatinolytic activities of secreted MMP-2 in a dose-dependent manner which corresponded to the inhibition of EC invasion. 

In addition, we examined the expression of adhesion and invasion related factors, including integrin *β*5, ESM-1, and ICAM-1 *in vivo*. The whole cell staining intensities of integrin *β*5 and ESM-1 protein were markedly reduced in TET treated tumors, compared with those of the tumors from the control group (Figure 2(e)). On the contrary, the ICAM-1 level in cell was significantly decreased by TET treatment (Figure 2(e), the middle column). These results have demonstrated that TET can effectively inhibit EC adhesion, migration, and invasion through mechanisms of changing the integrin *β*5, ESM-1, and ICAM-1 expressions.

### 3.3. TET Inhibits Tubeformation of HUVECs

**Figure 3:**
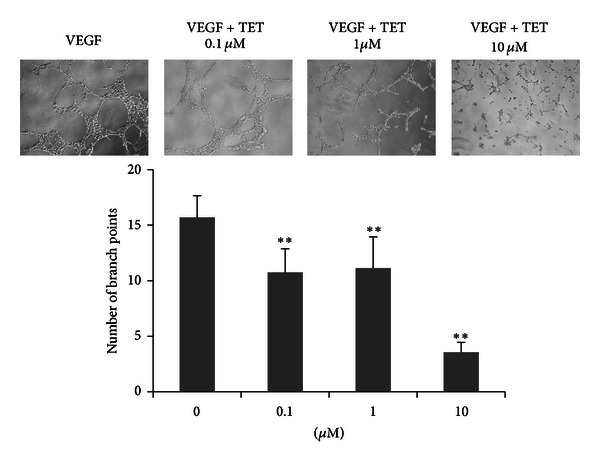
TET inhibits tube formation in HUVECs. The effects of TET on HUVEC differentiation were examined by their *in vitro* tube formation ability on matrigel. The branch points of the capillary-like tubes were counted under light microscopy (magnification 100x). Each value represents the mean ± S.D. of triplicate samples in each case. **P < 0.01 versus the VEGF-only group.

The effect of TET on the capillary tube formation of ECs was examined. In the absence of VEGF, there was no tube network structure in ECs, whereas the addition of VEGF (20 ng/mL, positive control) induced the formation of tube or cordlike structure and tube network on GFR matrigel. Cultured with TET resulted in shorter and less blunted tubes of ECs than those of VEGF control group (Figure 3(a)). Quantitative measurements showed that TET caused an increase in mean tube branch point formation as compared to VEGF 20 ng/mL group (positive control) (Figure 3(b)). The number of branch was reduced from 15.7 per area in the control group to 3.6 in the group treatment with TET (10 *μ*M) (*P* < 0.01).

### 3.4. TET Inhibits *In Vivo* Tumor Metastasis in Mouse Breast Tumor Model

**Figure 4:**
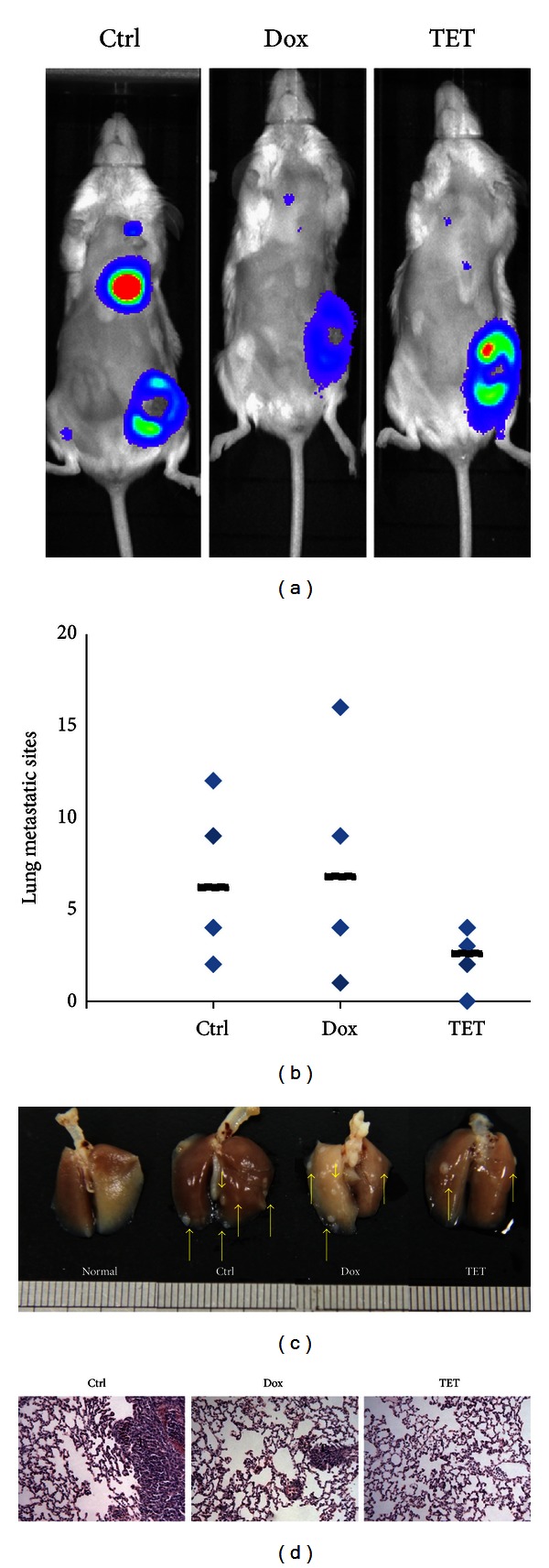
TET inhibits *in vivo* tumor metastasis in mouse breast cancer model. (a) Bioluminescence imaging of TET treated tumor bearing mice. Firefly luciferase-tagged 4T1 cells were injected into the MFP of BALB/c mice and TET was orally administered (10 mg/kg body weight, once every two days); images were obtained by using IVIS 200 imaging system. Representative Xenogen imaging results at week 4 are shown. (b) TET reduced tumor lung metastasis sites in 4T1 tumor-bearing mice. Picture shows the number of lung metastasis sites on the lung surface for each mouse. The black line shows the average number of metastasis sites for each group. (c) Photograph of pulmonary metastases. Animals were sacrificed after 4 weeks, and the lungs were dissected and photographed. (d) Histological examination of lung samples. Lung tissues sections were stained with H&E stain, and photographs were made under a microscope at a magnification of 400x (T: tumor).

We next investigated the *in vivo* antimetastatic activity of TET using a mouse breast cancer model. Briefly, exponentially growing firefly luciferase-tagged 4T1 cells were injected into the MFP of BALB/c mice, and TET was orally administered (10 mg/kg body weight, once every two days). As shown in Figure 4(a), the Dox and TET treatment groups exhibited significantly decreased Xenogen imaging signal in lung, when compared with the control group four weeks after treatment. At sacrifice, lung metastases were counted. In keeping with the *in vitro* data, the number of metastasis sites on the lung surface was remarkably decreased by TET, from 6.2 to 2.6 for each mouse, rather than that of Dox treated mouse (Figures 4(b) and 4(c)). Moreover, histological analysis (H & E staining) indicated that TET treatment group exhibited a decreased metastatic tumor mass in lung (Figure 4(d)).

### 3.5. TET Inhibits *In Vivo* Angiogenesis in 4T1 Tumor Bearing Mice

**Figure 5:**
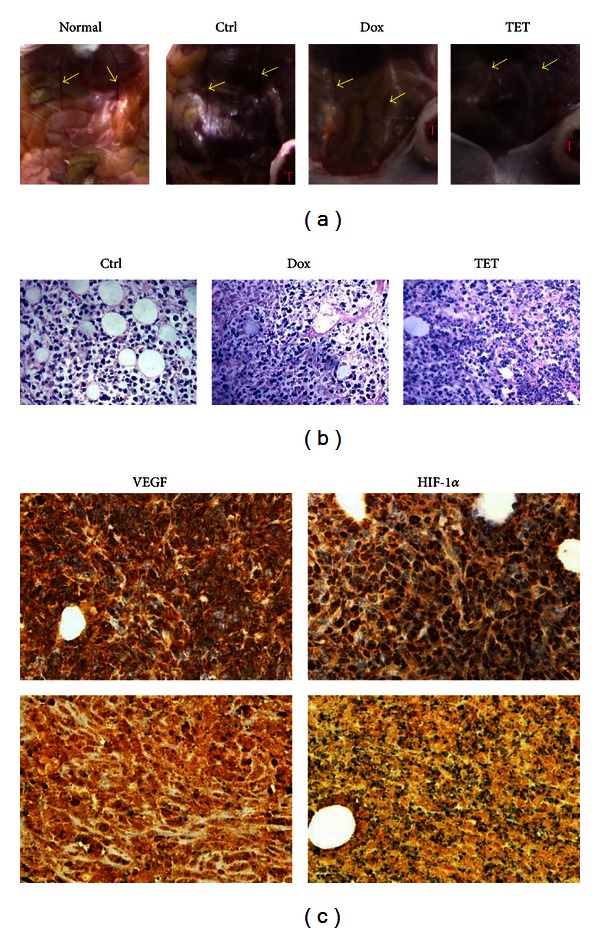
TET suppressed tumor angiogenesis in 4T1 tumor bearing mice. (a) The blood vessel diameter of TET treated tumor bearing mice. Picture shows the diameter of blood vessel on mice abdomen for each mouse. (b) Hematoxylin & Eosin staining of tumor tissues. Retrieved tumor samples were fixed, embedded, and subjected to H&E staining. Representative images are shown (magnification, ×400). (c) VEGF and HIF-1*α* expressions in tumor tissues. Representative staining results are shown: 4T1 tumor-bearing BALB/c mice (top panel) and TET treated mice (bottom panel) (magnification, ×400).

The effect of TET on angiogenesis *in vivo* was also examined in the animal model. As shown in Figure 5(a), four weeks after MFP injection of 4T1 cells into mice, the diameter of the blood vessels in the tumor implanted side is increased, rather than in the other side, but TET could significantly inhibit the increase of the blood vessel diameter from control levels. Consistent with gross observations, solid tumor sections further indicated that TET inhibited neovascularization in tumor mass *in vivo*, and the average number of new capillaries blood vessel in control group was more than that in TET treated group (Figure 5(b)). These results demonstrated that TET is a potent inhibitor of vascularization and angiogenesis.

We sought to further investigate the mechanism behind the TET mediated inhibition of angiogenesis activity. As shown in Figure 5(c), we examined the expression of metastatic and angiogenic related proteins, including VEGF and HIF-1**α* in vivo*. The whole cell and nuclear staining intensities of VEGF and HIF-1*α* were markedly reduced in TET treated tumors, compared with those of the control group.

**Figure 6:**
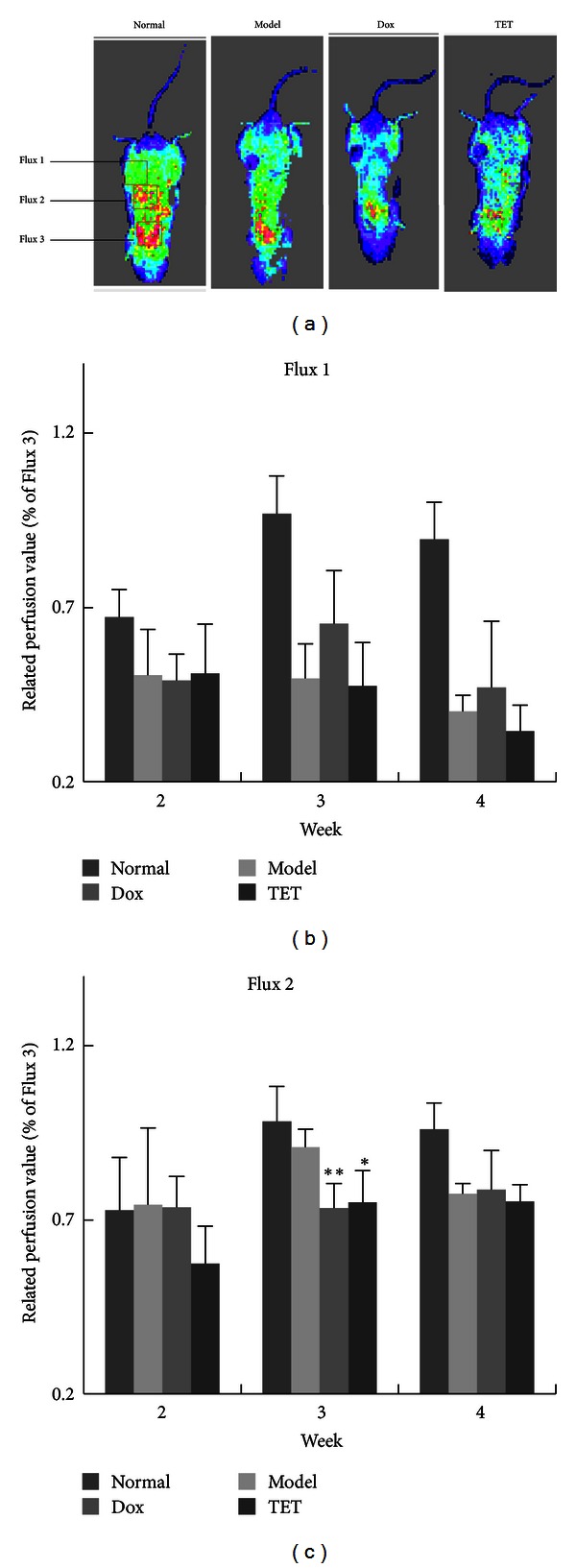
TET inhibits *in vivo* blood perfusion in 4T1 tumor bearing mice. (a) *In vivo* LDPI imaging of mice. TET was orally administered (10 mg/kg, once/2 days). With a distance of 10 cm between the scanner and the skin surface, three examined areas (1.4 ∗ 1.4 cm) covered the tumor (Flux 1), adjacent healthy skin around tumor (Flux 2), and the heart of mice (Flux 3). (b and c) Quantitative analysis of microvascular blood perfusion in mice. Related perfusion (Flux 1) = *F*1/*F*3∗100%; related perfusion (Flux 2) = *F*2/*F*3∗100%. *P < 0.05, **P < 0.01 versus vehicle control.

Lastly, to investigate the blood perfusion change of TET treated tumor bearing mice in tumor surface and skin around tumor area, we took advantage of the laser Doppler perfusion imaging (LDPI) to measure the local perfusion pattern. In the LDPI, the related perfusion of the mice abdomen was decreased after tumor being implanted for 3 weeks, especially the tumor site (Figure 6(a)). The related perfusion of tumor site (Flux 1) in TET group was decreased to 49% lower than that in healthy mice, but with no difference from that of the model group. It suggested that TET could slightly improve the necrosis of tumor 4 weeks after TET administration (Figure 6(b)). However, the related perfusion around tumor site (Flux 2) in TET group was significantly decreased to 82.54% of the model group (*P* < 0.05), which indicated that TET could markedly decrease the local blood perfusion of tumor 3 weeks after TET administration (Figure 6(c)) and suggested that the tumor angiogenesis was suppressed by TET treatment. 

Taken together, these *in vivo* results strongly suggest that TET may inhibit the tumor metastasis of breast cancer, possibly by reducing angiogenesis activity and related protein level of breast cancer cells, although further investigation is required.

## 4. Discussion

The growth and progression of solid tumors are usually limited by the nutrient supply for tumor. Thus, the blockage of microvessels formation and local blood perfusion in tumor might be useful in cancer therapy. Recently, more than 20 antiangiogenic drugs including TNP-470, thalidomide, and endostatin are subjected to different phases of clinical trials. In addition, phytochemicals such as resveratrol, salvianolic acid B, and ginseng saponins were found to exert inhibitory effect on the vascularization [22].

TET has been shown to exhibit anticancer activity in many *in vivo* models [5, 9, 10]. TET-treated mice (10 mg/kg/day) have fewer metastases than vehicle treated mice, and no acute toxicity or obvious body weight changes [23]. Recent studies showed that TET induces cell cycle arrest and also induces apoptosis in many human cancer cells. In our previous study, we found that inhibition of Wnt/beta-catenin signaling might contribute to the anticancer effects of TET [9]. Nonetheless, it is conceivable that other signaling pathways may also participate in TET's anticancer activity. For example, activation of glycogen synthase kinase 3*β* (GSK-3*β*), generation of ROS, activation of p38 mitogen-activated protein kinase (p38 MAPK), and upregulation of p53, p21, p27, and Fas might contribute to the anticancer effects of TET [5, 24–29].

As mentioned previously, TET exhibits significant anticancer activity both *in vitro* and *in vivo*, as well as its inhibitory effect on tumor metastasis and angiogenesis. Chen et al. found that TET inhibits the expression of VEGF in glioma cells, has cytotoxic effect on ECV304 HUVECs, and suppresses *in vivo* angiogenesis in rat [10]. However, the tumor related angiosuppressive property of TET and the molecular mechanism that underlies its activity are not fully understood. In the present study, we used a new LDPI method combined with different angiogenesis assays that are related to proliferation, adhesion, migration, invasion, and tube formation of EC during angiogenic process, to assess the angiosuppressive activity of TET.

Results from the present study demonstrated that TET exerted inhibitory effect on proliferation, adhesion, and capillary tube formation of ECs in a dose-dependent manner. Interestingly, the blood perfusion of the periphery of tumors was significantly reduced by TET treatment. Owing to that the blood perfusion is usually proportional to the body's blood vessels density [30], this result implied that the antimetastasis effect of TET was passably related to the angiosuppressive activity. Similar cases were also observed in the tumor mass of TET treated 4T1-tumor bearing mice. TET was found to effectively suppress the formation of micro vessels in tumor. Furthermore, since tube formation of HUVEC involves EC attachment, migration, and production of ECM degrading enzymes, data in the presented paper indicated that all these steps were interfered by TET and resulted in the attenuation of angiogenesis *in vitro* and *in vivo*. Thus, TET may be useful in cancer metastasis by acting as a specific and effective angiosuppressive agent.

We investigated the molecular mechanism underlying the antivascularization activity of TET in breast cancer. TET treated group exhibited a decreased level of VEGF, HIF-1*α*, ESM-1 and Integrin *β*5 protein but upregulation of ICAM-1 level. These *in vivo* results strongly suggest that the inhibitory effect of TET on breast cancer metastasis may be at least in part mediated by inhibiting tumor angiogenesis factors (VEGF and HIF-1*α*) or regulating adhesion factors (Integrin *β*5, ESM-1, and ICAM-1), although further investigation is required.

One of the key cell signaling pathways involved in cancer tumorigenesis and metastasis is the hypoxic pathway. As we know, HIF-1*α* binds to HREs and induces subsequent expression of genes encoding angiogenic factors, such as VEGF and MMPs, leading to angiogenesis [31]. TET could reduce the expression of HIF-1*α* and then decrease VEGF level and MMPs activity. It is generally believed that VEGF could activate ECs; activated ECs produce many types of enzymes such as matrix metalloproteinases (MMPs) that break down the stroma and ECM proteins [32]. This is the critical step in angiogenesis as well as metastasis. The invasion assay involving the migration of HUVEC through ECM (matrigel) demonstrated that TET could reduce the chemoinvasive ability of EC by reducing the gelatinases activities of the cell culture medium. These noteworthy results indicated that the angiosuppressive effect of TET could be possibly due to the reduction of HIF-1*α* and/or VEGF expression, as well as MMPs activities.

Recently, several research groups have tried to identify cell adhesion suppressors which could inhibit cancer metastasis by blocking the lodging in blood vessels in the distant organs of disseminated cancer cells or cell clusters [33]. Integrins are important mediators of the malignant phenotype during oncogenic transformation [34]. Breast carcinoma cells express high levels of integrin *β*5. Bianchi-Smiraglia et al. indicated that cells deficient in integrin *β*5 have lower migration and proliferative capacities, and ERK signaling pathway plays an important role in the function of integrin *β*5 in cancer [35]. Our results show that the expression of integrin *β*5 was decreased by TET treatment and accompanied by a reduced phosphorylated-ERK level. On the other hand, the adhesion factor ESM-1, which could inhibit leukocyte adhesion and migration through the endothelium, was increased in tissue and serum from colorectal cancer patients and ESM-1 silencing decreased cell survival, migration, and invasion and modulated cell cycle progression in hepatocellular carcinoma [36, 37]. We notified that TET also decreases ESM-1 expression in breast cancer cells, which leads to an inhibition effect of tumor metastasis. Taken together, TET suppressed the integrin *β*5 and ESM-1 expressions, then depressed the activation of ERK, and regulated cellular proliferation, adhesion, and survival in ECs.

**Figure 7:**
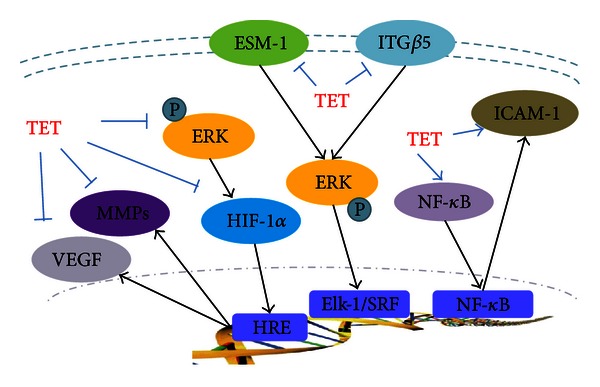
The influence of TET on the angiogenesis and metastasis related factors.

Another important adhesive factor involved in TET's angiosuppressive effect is ICAM-1; several researches showed that ICAM-1 synthesis in ECs is regulated by activation of p38 and NF-*κ*B [38, 39], and ICAM-1 could induce cell adhesion by active ERK, JNK, and p38 pathways [40, 41]. Our IHC results present an upregulation of ICAM-1 in tumor tissue, along with the increasing of NF-*κ*B, and suggested that the promotion effect of TET on NF-*κ*B and ICAM-1 expressions is closely related to its antiangiogenesis effects. Taken together, as shown in Figure 7, our studies indicate that TET is a potential inhibitor of tumor angiogenesis and metastasis by targeting the angiogenesis and metastasis related factors.
